# Supplementary material for: Is there meaningful influence from situational and environmental factors on the physical and technical activity of elite football players? Evidence from the data of 5 consecutive seasons of the German Bundesliga
Source: PLoS One. 2021 Mar 9;16(3):e0247771. doi: 10.1371/journal.pone.0247771 (PMC7943014; doi:10.1371/journal.pone.0247771)
Supplement: Attachment — Submitted filename: Responses for Reviewers PLOS.docx [file pone.0247771.s001.docx]

Reviewer 1

In response to your comments in the review of the manuscript PONE-D-20-32150 "Is there meaningful influence from situational and environmental factors on the physical and technical activity of elite football players? Evidence from the data of 5 consecutive seasons of the German Bundesliga", we would like to inform you about corrections made and augmentations provided.

Reviewer #1: 1. Please correct the abbreviation WGBT (Wet Bulb Globe Temperature)

**The correction has been made.**

2. Please provide the ethical reference number from the local Board of Ethics (pg5)

**The correction has been made.**

3. Please justify that the sample size in this study was enough based on the holistic approach to monitoring and larger sample size as stated on the page 3

**a) We thank the Reviewer for this remark. In the opinion of the authors, a research sample containing over 21,000 authorizes a holistic approach. The analysis divided into players positions shows that each one contains over 2,600 observations, which is definitely more than in other publications of similar type. For example, on the evolution of football**

**- Barnes C, Archer DT, Hogg B, Bush M, Bradley PS. The evolution of physical and technical performance parameters in the English Premier League. Int J Sports Med. 2014 Dec; 35(13): 1095-100.**

4. Please state how the number of 779 players with 21,971 individual match observations were considered in the analysis (CD n=6187, FB n=4806, CM=4798, WM n=3545, FW n=2635). Were there any changes in the players' positions in each match or other matches?

**We thank the Reviewer for this remark. Yes, the players’ positions were sometimes changed from match to match. But according to the data provider, within one match, the playing position of each player is assigned by his club at the start of the game, and would not have been altered (Definitionskatalog Offizielle Spieldaten – Bundesliga website - https://s.bundesliga.com/assets/doc/10000/2189_original.pdf)**

5. Please consider to apply the right justify for the values in the Table 2

**The correction has been made.**

6. It is more meaningful to have the minimum and maximum values in the Figures. eg the highest and lowest temperature, humidity etc.

**We thank the Reviewer for this remark. The data presented in the Figures were effect data. The minimum and maximum values of temperature and humidity were descriptive data, which have been added into the revised table 2.**

We would like to express our sincere thanks to the Reviewers for their time and valuable comments on our study. We trust that the corrections and revisions made will improve the editorial value and content of our study and allow us have our work published in your renowned journal.

Sincerely yours,

The Authors

Reviewer 2

In response to your comments in the review of the manuscript PONE-D-20-32150 "Is there meaningful influence from situational and environmental factors on the physical and technical activity of elite football players? Evidence from the data of 5 consecutive seasons of the German Bundesliga", we would like to inform you about corrections made and augmentations provided.

Reviewer #2: line 109: WGBT (Wet Bulb Globe Temperature) index should be written as " Wet Bulb Globe Temperature (WBGT) index

**The correction has been made.**

Lines 142-145 should be moved to the descriptive part of the results section. These abbreviations should have been written in full before being used (CD , FB , CM 143 ,WM , FW)

**The correction has been made.**

Figures 2 to 4 should be made more viewable for reading by keeping at most 3 graphs per row.

**The authors tried to generate figures according to the reviewer's suggestions, but then the figures were less readable - it is better if all 5 items are on the horizontal axis and the next parameters are arranged in lines one below the other than vice versa. Due to the analysis of five player positions, many factors and the desire not to increase the number of figures, the authors after an in-depth discussion, decided to leave the figures in their current form**.

The authors should avoid section numberings

**The correction has been made.**

We would like to express our sincere thanks to the Reviewers for their time and valuable comments on our study. We trust that the corrections and revisions made will improve the editorial value and content of our study and allow us have our work published in your renowned journal.

Sincerely yours,

The Authors
